# *ACVR1B* rs2854464 Is Associated with Sprint/Power Athletic Status in a Large Cohort of Europeans but Not Brazilians

**DOI:** 10.1371/journal.pone.0156316

**Published:** 2016-06-02

**Authors:** Sarah Voisin, João Paulo F. L. Guilherme, Xu Yan, Vladimir P. Pushkarev, Pawel Cieszczyk, Myosotis Massidda, Carla M. Calò, Dmitry A. Dyatlov, Vitaliy A. Kolupaev, Yuliya E. Pushkareva, Agnieszka Maciejewska, Marek Sawczuk, Antonio H. Lancha, Guilherme G. Artioli, Nir Eynon

**Affiliations:** 1 INRA, UMR1198 Biologie du Développement et Reproduction, F-78350, Jouy-en-Josas, France; 2 Institute of Sport, Exercise and Active Living (ISEAL), Victoria University, Victoria, Melbourne, Australia; 3 School of Physical Education and Sport, University of Sao Paulo, Sao Paulo, Brazil; 4 Laboratory of Radiation Genetics, Urals Research Centre for Radiation Medicine of the Federal Medical-Biological Agency of Russia, Chelyabinsk, Russia; 5 Academy of Physical Education and Sport, Department of Tourism and Recreation, Gdansk, Poland; 6 Department of Life and Environmental Sciences, University of Cagliari, Cagliari, Italy; 7 Ural State University of Physical Culture, Chelyabinsk, Russia; 8 South Ural State Medical University, Chelyabinsk, Russia; 9 Department of Physical Culture and Health Promotion, University of Szczecin, Szczecin Poland; 10 Department of Radiation Biology, Chelyabinsk State University, Chelyabinsk, Russia; Cleveland Clinic, UNITED STATES

## Abstract

Skeletal muscle strength and mass, major contributors to sprint/power athletic performance, are influenced by genetics. However, to date, only a handful of genetic variants have been associated with sprint/power performance. The *ACVR1B* A allele (rs rs2854464) has previously been associated with increased muscle-strength in non-athletic cohort. However, no follow-up and/or replications studies have since been conducted. Therefore, the aim of the present study was to compare the genotype distribution of *ACVR1B* rs2854464 between endurance athletes (E), sprint/power (S/P) athletes, mixed athletes (M), and non-athletic control participants in 1672 athletes (endurance athletes, n = 482; sprint/power athletes, n = 578; mixed athletes, n = 498) and 1089 controls (C) of both European Caucasians (Italian, Polish and Russians) and Brazilians. We have also compared the genotype distribution according to the athlete’s level of competition (elite vs. sub-elite). DNA extraction and genotyping were performed using various methods. Fisher's exact test (adjusted for multiple comparisons) was used to test whether the genotype distribution of rs2854464 (AA, AG and GG) differs between groups. The A allele was overrepresented in S/P athletes compared with C in the Caucasian sample (adjusted p = 0.048), whereas there were no differences in genotype distribution between E athletes and C, in neither the Brazilian nor the Caucasian samples (adjusted p > 0.05). When comparing all Caucasian athletes regardless of their sporting discipline to C, we found that the A allele was overrepresented in athletes compared to C (adjusted p = 0.024). This association was even more pronounced when only elite-level athletes were considered (adjusted p = 0.00017). In conclusion, in a relatively large cohort of athletes from Europe and South America we have shown that the *ACVR1B* rs2854464 A allele is associated with sprint/power performance in Caucasians but not in Brazilian athletes. This reinforces the notion that phenotype-genotype associations may be ethnicity-dependent.

## Introduction

Skeletal muscle strength and mass are major contributors to elite athletic performance in a variety of sport disciplines, especially those where explosive muscle contractions are critical (e.g. sprint/power oriented disciplines) [1]. There is considerable variability in sprint, strength, and power performance within athletes of similar age, body composition, and training history. One possible explanation for this variability is the athletes’ genetic makeup [2]. The current estimated heritability for muscle strength and muscle mass ranges from 31% to 78%, with large differences between muscle groups, contraction velocities and muscle lengths [3,4].

To date, only a handful of genetic variants have been robustly associated with sprint/power performance. Two candidate gene variants associated with sprint/power performance with reasonable replication in different groups of elite sprint/power athletes are the *ACTN3* R577X [5,6] and the *ACE* I/D [7,8]. We have also recently shown that variants in the *EPAS1* [9] and the *MCT1* genes [10] are associated with sprint/power performance in European athletes. However, these studies require replication and verification in larger cohorts, and the consensus shared between scientists is that there are many other undiscovered variants associated with sprint/power performance.

A gene variant (rs2854464) within the Activin A Receptor type 1b gene (*ACVR1B*) has previously been associated with muscle-strength phenotypes [11]. Initial studies using microsatellite markers and linkage analysis have identified linkage peaks associated with muscle strength in the 12q12-14 chromosomal region [12,13]. The same research group has then used different approaches to show that the *ACVR1B* rs2854464 A allele is associated with increased muscle strength in healthy, non-athletic individuals [11]. To date, no follow-up and/or replications studies have been conducted to confirm or refute these findings. Therefore, whether this gene variant influence athletic performance and/or gains in muscle mass and strength in either elite athletes or the general population remains unclear.

One of the major drawbacks in the field of Sports Genomics is the relatively low sample of elite athletes and collaborative effort is required to move the field forward, and enhance our understanding of the genes that influence athletic performance. The Athlome Project Consortium [14] has been therefore recently established, and one of its aims is to identify gene variants that contribute to elite performance in large-scale, collaborative efforts involving cohorts from different countries.

Therefore, the present study aimed to compare the genotype distribution of *ACVR1B* rs2854464 between endurance athletes, sprint/power athletes, and non-athletic control participants in a large cohort (n = 1672 athletes, and n = 1089 controls) of both European Caucasians (Italian, Polish and Russians) and Brazilians. Furthermore, the association between rs2854464 and athletic status (i.e., ‘elite’ and ‘sub-elite’ level) was also examined. In light of the previously observed association between the *ACVR1B* A allele and muscle strength, we hypothesized that the A allele would be associated with elite sprint/power performance.

## Material and Methods

The study was conducted according to the Declaration of Helsinki. All participants have signed an informed consent form prior to the study. The study was approved by the ethics committees of the University of Sao Paulo, Brazil, the Pomeranian Medical University, Poland, the University of Cagliari, Italy and the Ural State University of Physical Culture, Russia. The full dataset used in this study can be found in S1 Table.

### Participants

A total of 1672 athletes (endurance athletes, n = 482; sprint/power athletes, n = 578, mixed athletes, n = 498), and 1089 non-athletic controls volunteered to participate in this study. Athletes and controls were from four countries: Brazil (n_controls_ = 257, n_athletes_ = 474), Italy (n_controls_ = 84, n_athletes_ = 125), Poland (n_controls_ = 500, n_athletes_ = 350), and Russia (n_controls_ = 248, n_athletes_ = 723). Participants from the European countries (i.e., Italy, Poland, and Russia) were self-reported unrelated Caucasians for ≥ three generations, whereas the Brazilian athletes have been treated as a different group given the admixture in the Brazilian population (see below in ‘population stratification’ section). Athletes were classified as endurance (E), sprint/power (S/P) or mixed (M) athletes according to the characteristics of their sports disciplines (i.e., distance, duration and metabolic requirements) (Table 1). When categorization was not straightforward (e.g. if a runner, for example, was engaged in both the 800m (M) and 1500m (E) distances), we classified the athlete as uncertain (U). All athletes were in the top 10 national rank in their sports discipline and grouped as ‘elite level’ or ‘sub-elite (national) level’ according to individual’s best personal performance, as previously described [9,15]. Athletes in the elite group had participated in international competitions (e.g., World and Continental Championships, and/or Olympic Games), whilst those in the sub-elite group had only participated in national-level competitions.

**Table 1:** Classification of the athletes' disciplines.

Endurance (E)	Sprint/power (S/P)	Mixed (M)	Uncertain (U)
Biathlon	Archery	Badminton	Running (800-1500m)
Canoeing marathon	Artistic gymnastics	Bandy	Speed skating (500-3000m)
Cross-country skiing	Wrestling	Boxing	Speed skating (500-5000m)
Cycling endurance	Canoeing speed	Canoeing (200-1000m)	Speed skating (500-10000m)
Marathon	Cycling (1000m)	Decathlon	Speed skating (1500-3000m)
Mountain cycling	Cycling (2000m)	Fencing	Speed skating (1500-5000m)
Racewalking (20000m)	Discus throw	Figure skating	Speed skating (3000-5000m)
Road cycling	Diving	Futsal	Swimming (100-200m)
Rowing (2000m)	Gymnastics	Handball	Swimming (200-400m)
Rowing (5000m)	Hammer throw	Heptathlon	
Rowing (2000-10000m)	High jump	Ice hockey	
Running (1500m)	Javelin throw	Judo	
Running (3000m)	Jump	Karate	
Running (1500-3000m)	Jump/Running (100-200m)	Kickboxing	
Running (5000m)	Long jump	Pentathlon	
Running (1500-5000m)	Mogul skiing	Rhythmic Gymnastics	
Running (5000-10000m)	Pole vault	Running (800m)	
Running (>10000m)	Powerlifting	Soccer (midfielder)	
Shooting	Rowing (200-500m)	Speed skating (3000m)	
Speed skating (5000m)	Rowing (200-1000m)	Swimming (200m)	
Speed skating (5000-10000m)	Rowing (500m)	Taekwondo	
Speed skating (10000m)	Rowing (1000m)	Volleyball	
Speed skating stayer	Running (100m)	Water polo	
Steeple-chase	Running (200m)		
Swimming (400-800m)	Running (100-200m)		
Swimming (800m)	Running (400m)		
Swimming (800-1500m)	Running (100-400m)		
Swimming (1500m)	Shot put		
Swimming (>5000m)	Skating		
Triathlon	Ski-cross		
Walking	Ski jumping		
	Slalom skiing		
	Slopestyle		
	Snowboard-cross		
	Soccer (defender)		
	Speed skating (500m)		
	Speed skating (500-1000m)		
	Speed skating (500-1500m)		
	Speed skating (1000m)		
	Speed skating (1500m)		
	Speed skating (1000-1500m)		
	Swimming (50m)		
	Swimming (50-100m)		
	Swimming (100m)		
	Throw		
	Weightlifting		

#### Exclusion criteria

We have excluded from the analysis: athletes that had only participated in regional competitions (n = 7, all Brazilian); athletes whose genotype was undetermined (n = 2, all Polish); athletes that were of non-European origin in the Italian, Polish or Russian samples (n = 6, all Italian).

#### Population stratification

The Brazilian population is formed by extensive admixture between Amerindians, Europeans and Africans, and is one of the most variable populations in the world. Despite positive assortative mating by ancestry [16], self-reported ancestry remains an unreliable criteria for the Brazilian population [17–19]. Thus, we have treated the Brazilian cohort separately, regardless of their self-reported ethnicity.

### Genotyping

#### Brazilian sample

Genomic DNA was isolated from buccal epithelium obtained from mouthwashes. DNA was then extracted using chloroform, precipitated using ethanol and resuspended with 1x Tris-EDTA (Invitrogen). DNA quantification and quality assessment were performed using spectrophotometer (NanoDrop 2000, Thermo Scientific). Genotyping of the *ACVR1B* rs2854464 polymorphism was performed by using a pre-designed specific TaqMan^®^ SNP Genotyping Assays (ID: C__15826374_10, Applied Biosystems, Foster city, CA, USA), run and read performed in a Rotor Gene-Q real-time termocycler (Qiagen, Valencia, CA, USA), using 15 ng of the DNA samples and appropriate primers fluorescently labeled (FAM and VIC) MGB™ probes according to the manufacturer's instructions. A scatter plot was used to plot the final end data points and thus discriminating the alleles.

#### Italian sample

Genomic DNA was isolated from buccal swab, and extracted using Qiamp minikit according to the manufacturer's instructions. All samples were amplified with a classical PCR (Applied Biosystem) using the following primers: FORWARD-GCTTGCTGGTGCCTCTTTTC; REVERSE-CTTCACATTCCTCGGCCCTT. PCR products were sequenced by Macrogen through forward primers. For replication and genotype verification, 10% of samples were genotyped in duplicates.

#### Polish sample

Buccal epithelium was used to isolate genomic DNA with the GenElute Mammalian Genomic DNA Miniprep Kit (Sigma, Hamburg, Germany). In all the cohorts, genotypes were determined in duplicates using a Real-Time PCR-based allelic discrimination system (CFX96, Bio-Rad, USA) with Taqman probes. To discriminate *ACVR1B* rs2854464 alleles, TaqMan Pre-Designed SNP Genotyping Assays were used, similarly to those used in the Brazilian cohort.

#### Russian sample

Buccal epithelium or peripheral blood was used to isolate genomic DNA with the GeneJET™ Genomic DNA Purification Kit (Thermo Fisher Scientific Inc).

Genotyping of the *ACVR1B* rs2854464 polymorphism was performed with a TaqMan^®^ SNP genotyping assay similar to the assay used in the Brazilian and Polish cohort. K562 DNA High Molecular Weight from Promega Corp. (Cat # DD2011, Madison, WI, USA) served as a positive control sample. The *ACVR1B* rs2854464 genotype of the K562 DNA was A/G.

### Statistical analyses

#### Hardy—Weinberg equilibrium (HWE)

*χ*^2^ analysis was used to confirm whether the control group from each of the four samples met HWE expectations.

#### Distribution of rs2854464 genotypes between groups

Fisher's exact test was used to test whether the rs2854464 genotype distribution (AA, AG and GG) differs between groups. To eliminate the possibility of false positive results, p-values were adjusted for multiple comparisons as proposed by Benjamini and Hochberg [20]. Significance was set at p < 0.05.

## Results

### HWE and genotype distribution in the control sample

The genotype distribution of all control groups were in HWE (*χ*^2^ test, all p > 0.05). Moreover, the Italian, Polish and Russian controls had similar genotype distributions (Fisher's exact test, all p > 0.05, S1 Fig). Thus, to increase statistical power and to reduce the number of performed tests, we pooled the Italian, Polish and Russian samples together and referred to this sample as the "Caucasian sample". Genotype distributions of the individual cohorts are available in S1 Fig and Table 2.

**Table 2:** Genotype distributions in the four studied samples.

	**Brazilians**			**Brazilians elite**		
	AA	AG	GG	AA	AG	GG
**Control**	111 (43%)	107 (42%)	39 (15%)	111(43%)	107 (42%)	39 (15%)
**Endurance**	100 (44%)	103 (46%)	23 (10%)	49 (43%)	54 (48%)	10 (9%)
**Sprint/power**	57 (32%)	97 (54%)	26 (14%)	41 (37%)	56 (50%)	15 (13%)
**Mixed**	0	0	0	0	0	0
**Uncertain**	28	23	10	9	9	4
**All athletes**	185 (40%)	223 (48%)	59 (12%)	99 (40%)	119 (48%)	29 (12%)
	**Italian Caucasian**			**Italian Caucasian elite**		
	AA	AG	GG	AA	AG	GG
**Control**	42 (50%)	36 (43%)	6 (7%)	42 (50%)	36 (43%)	6 (7%)
**Endurance**	14 (58%)	8 (33%)	2 (9%)	11 (52%)	8 (38%)	2 (10%)
**Sprint/power**	35 (49%)	32 (45%)	4 (6%)	22 (51%)	19 (44%)	2 (5%)
**Mixed**	11 (52%)	9 (43%)	1 (5%)	9 (47%)	9 (48%)	1 (5%)
**Uncertain**	0	1	0	0	0	0
**All athletes**	60 (51%)	50 (43%)	7 (6%)	42 (51%)	36 (43%)	5 (6%)
	**Polish Caucasian**			**Polish Caucasian elite**		
	AA	AG	GG	AA	AG	GG
**Control**	263 (53%)	187 (37%)	50 (10%)	263 (53%)	187 (37%)	50 (10%)
**Endurance**	54 (56%)	40 (41%)	3 (3%)	54 (56%)	40 (41%)	3 (3%)
**Sprint/power**	55 (53%)	42 (40%)	7 (7%)	55 (53%)	42 (40%)	7 (7%)
**Mixed**	91 (62%)	53 (36%)	3 (2%)	91 (62%)	53 (36%)	3 (2%)
**Uncertain**	0	0	0	0	0	0
**All athletes**	200 (57%)	135 (39%)	13 (4%)	200 (57%)	135 (39%)	13 (4%)
	**Russian Caucasian**			**Russian Caucasian elite**		
	AA	AG	GG	AA	AG	GG
**Control**	126 (51%)	102 (41%)	20 (8%)	126 (51%)	102 (41%)	20 (8%)
**Endurance**	72 (53%)	50 (37%)	13 (10%)	19 (54%)	14 (40%)	2 (6%)
**Sprint/power**	133 (59%)	84 (38%)	7 (3%)	56 (62%)	30 (33%)	4 (5%)
**Mixed**	188 (57%)	119 (36%)	23 (7%)	75 (60%)	48 (38%)	3 (2%)
**Uncertain**	21	11	2	11	5	0
**All athletes**	414 (57%)	264 (37%)	45 (6%)	161 (60%)	97 (36%)	9 (4%)

### Endurance athletes vs. controls

**Fig 1:**
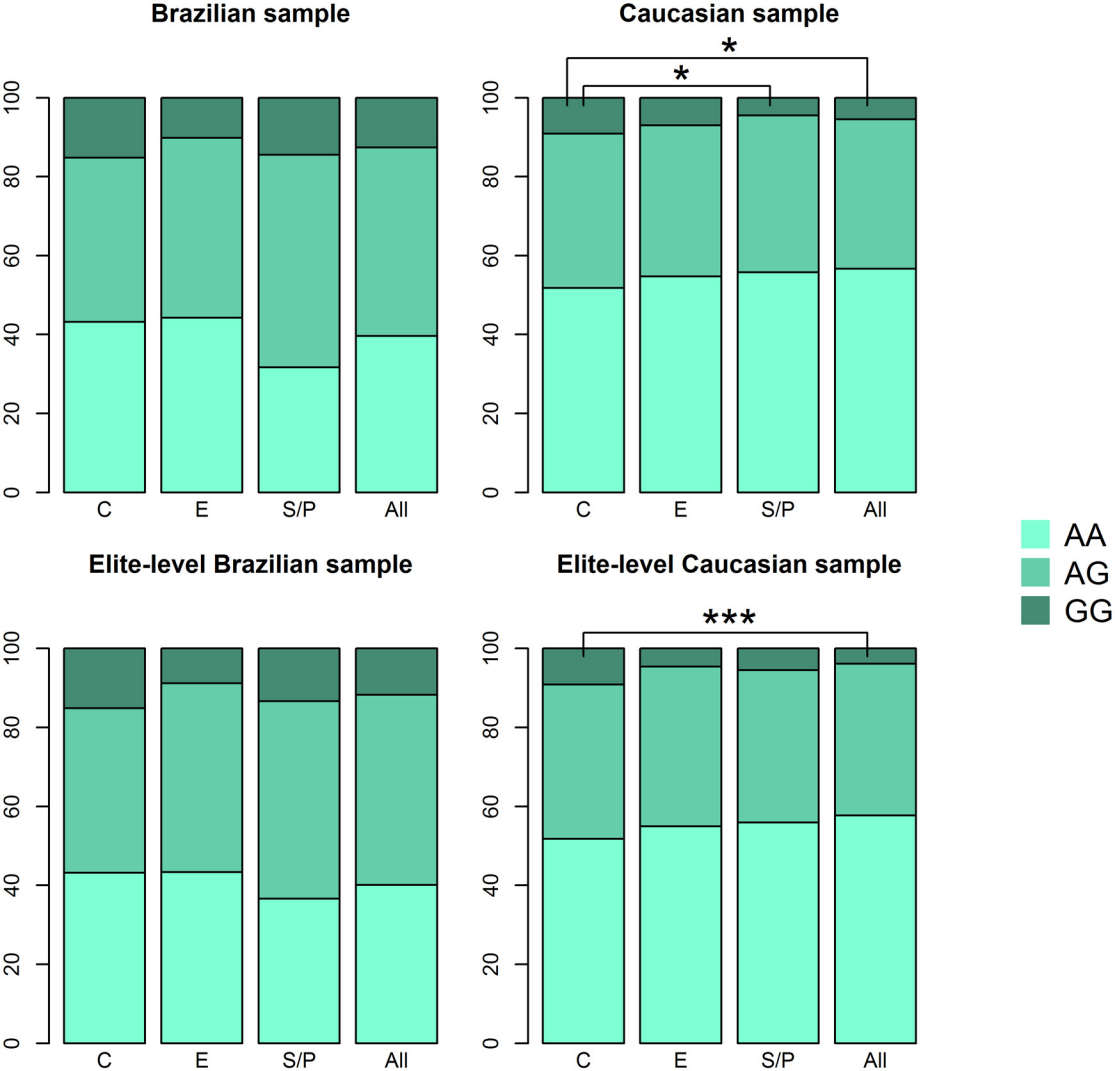
Genotype distributions at rs2854464 in Brazilian and Caucasian samples. C = controls; E = endurance athletes; S/P = sprint/power athletes; All = all athletes; *adjusted p-value < 0.05; **adjusted p-value < 0.01; ***adjusted p-value < 0.001.

There were no differences in genotype distribution between E athletes and C, in neither the Brazilian nor the Caucasian samples (Fisher's exact test, all adjusted p > 0.05, Table 2, Fig 1).

### Sprint/power athletes vs. controls

The A allele was overrepresented in S/P athletes compared with C in the Caucasian sample (Fisher's exact test, adjusted p = 0.048, Table 2, Fig 1). However, no difference was observed when comparing elite-level S/P athletes vs. C (Fisher's exact test, adjusted p = 0.17, Table 2, Fig 1).

In contrast to what was observed in the Caucasian sample, there was a trend towards an underrepresentation of the A allele in S/P athletes compared to C in the Brazilian sample. (Fisher's exact test, adjusted p-value = 0.058, Table 2, Fig 1).

### All athletes vs. controls

When comparing all Caucasian athletes regardless of their sporting discipline (E, S/P, M and U) to C, we found that the A allele was overrepresented in athletes compared to C (Fisher's exact test, adjusted p = 0.024, Table 2, Fig 1). This association was even more pronounced when only elite-level athletes were considered (Fisher's exact test, adjusted p = 0.00017, Table 2, Fig 1).

There were no differences in genotype distributions between all athletes and C in the Brazilians sample (Fisher's exact test, adjusted p = 0.35, Table 2, Fig 1).

## Discussion

Sprint/power performance, as well as muscle strength and mass are influenced by genetics [5]; yet, only a few genetic variants associated with either elite sprint/power performance and/or muscle strength and muscle mass have been identified to date. The rs2854464 polymorphism within the Activin A Receptor type 1b gene was recently shown to be associated with muscle strength in a properly-designed study [11]. In the present study, we sought to further explore the relevance of this polymorphism to sprint/power performance. We found that the *ACVR1B* rs2854464 is differently associated with sprint/power performance in a relatively large cohort (n = 1,672) of Caucasian and Brazilian athletes. While the A allele was overrepresented in Caucasian athletes, and more specifically in sprint/power Caucasian athletes compared with controls, there was a trend towards an underrepresentation of the A allele (p = 0.058 after multiple testing correction) in sprint/power Brazilian athletes. Our results reinforce the hypothesis that an association between any genetic variant and athletic performance might be dependent on the population’s ethnic background.

In the present study we have addressed some of the limitations inherent in previous elite athlete case-control studies. Firstly, we have studied four cohorts of elite and sub-elite athletes, including three European Caucasian athletes and a Brazilian cohort. Consequently, the number of athletes (n = 1,672) is significantly higher compared to previous genetic association studies, and demonstrates the benefits of a collaborative approach that has been recommended in the field of exercise genomics [21,22]. Secondly, previous reports have grouped together sprint and power athletes from mixed sports disciplines and events. Here, we have embraced a more stringent approach and divided the athletes to four categories, based on the physiological demands of each specific event (Table 1). Thirdly, to avoid being exposed to false positive results and as recently suggested [23], all p-values were adjusted for multiple comparisons.

There is a biochemical/cellular rationale to suggest that common variants within *ACVR1B* would be associated with sprint/power and/or strength performance. *ACVR1B* encodes the Activin A receptor type 1b protein, which is part of the TGF-β (Transforming Growth Factor-β) superfamily, a set of growth factors that regulates the expression level of several genes implicated in controlling muscle growth [24]. Myostatin is perhaps one of the most important members of the TGF-β; it down-regulates muscle mass during both pre- and post-natal stages [24]. Activin receptor type 2b (ACVR2B) is the primary type 2 receptor for myostatin. However, the type 1 receptor is important for the muscle signalling cascade following the interaction between myostatin and ACVR2B, being essential for the signal propagation through the plasma membrane. After binding of myostatin to ACVR2B, ACVR1B is recruited and contributes to the formation of a heteromeric active receptor complex [25]. According to Windelinckx et al. [11], “the rs2854464 polymorphism is located in a putative miR-24-binding site in the 3' untranslated region (UTR) of the ACVR1B mRNA”. There is evidence showing that miR-24 may decrease gene and protein expression of *ACVR1B* [26] and play a role in myoblast differentiation, inhibiting the skeletal muscle differentiation induced by TGF- β [27]. A potential explanation for the downstream association between rs2854464 A allele and sprint/power performance, is that it might provide a better affinity between the 3' UTR of *ACVR1B* mRNA and miR-24, leading to a more effective translational inhibition and decay of *ACVR1B* mRNA. It has been shown that pharmacological blockade of the activin A signalling pathway by using soluble activin type II receptors (ligand level), or antibody to ActRII (receptor level) increases muscle and bone mass, correct anaemia or protect against diet-induced obesity [28,29].

Using knock-out (KO) mice model, and multiple human association studies, the biochemical/cellular rationale that common genetic variants in muscle-related genes may influence sprint/power performance has been well demonstrated in the case of the *ACTN3* R577X variant, currently the most promising candidate gene to influence sprint/power performance [30,31]. However, this variant can explain only ~1.5% of the variance in elite sprint/power performance [6] and many other gene variants are still to be identified. Therefore, with the limited knowledge we currently hold, a recent consensus has stated that genetic tests are not yet valid for talent identification or for individualizing training prescription to optimise performance [32].

Here, we have identified for the first time an association between the *ACVR1B* rs2854464 and elite sprint/power athletic status in Caucasian athletes. We note that the next step required is to replicate these findings in other cohorts of elite athletes with different geographical backgrounds (similarly to *ACTN3* R577X). We also stress that to further confirm our findings, future studies should examine functional outcomes (i.e., causation) related to the effects of *ACVR1B* on muscle physiology. The recently launched Gene SMART (Skeletal Muscle Adaptive Response to Training) study, that aims to identify gene variants that predict skeletal muscle responses to High-Intensity Interval Training, might be useful to further confirm whether *ACVR1B* is indeed important to performance.

## Conclusion

In conclusion, in a relatively large cohort of athletes from Europe and South America, we have shown that the *ACVR1B* rs2854464 A allele is associated with sprint/power performance in Caucasians but not in Brazilians athletes. This reinforces the notion that phenotype-genotype associations may be ethnicity-dependent. We acknowledge that elite athletic performance is a polygenic trait [33]; therefore, more genetic variants influencing sprint/power performance are yet to be discovered.

## Supporting Information

S1 Fig Genotype distributions at rs2854464 in Italian, Polish and Russian samples. C = controls; E = endurance athletes; S/P = sprint/power athletes; All = all athletes.
(TIF)

S1 Table The full dataset used in this study. (XLSX)
