# Supplementary figures and images for: ACVR1B rs2854464 Is Associated with Sprint/Power Athletic Status in a Large Cohort of Europeans but Not Brazilians
Source: PLoS One. 2016 Jun 2;11(6):e0156316. doi: 10.1371/journal.pone.0156316 (PMC4890799; doi:10.1371/journal.pone.0156316)

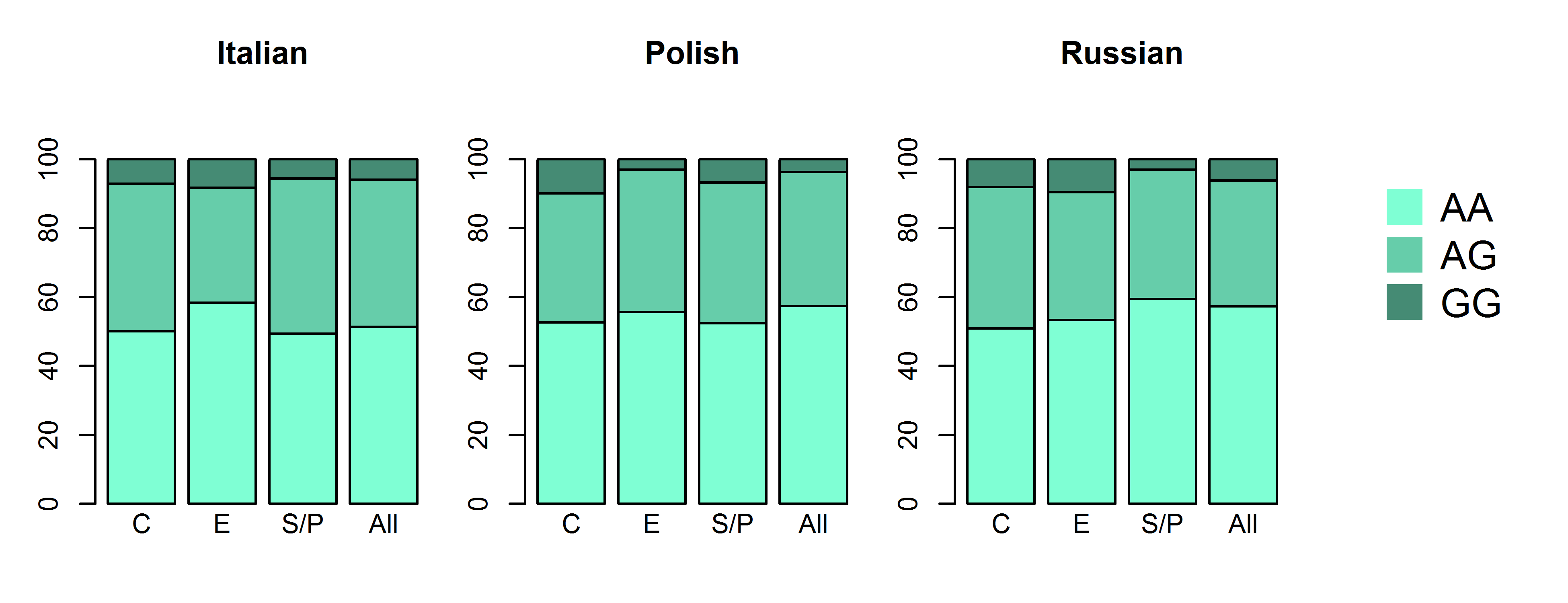

Supplement: S1 Fig — C = controls; E = endurance athletes; S/P = sprint/power athletes; All = all athletes. (TIF) [file pone.0156316.s001.tif]
